# Identification of *Hop stunt viroid* infecting *Citrus limon* in China using small RNAs deep sequencing approach

**DOI:** 10.1186/s12985-015-0332-2

**Published:** 2015-07-07

**Authors:** Xiu Su, Shuai Fu, Yajuan Qian, Yi Xu, Xueping Zhou

**Affiliations:** Institute of Biotechnology, State Key Laboratory of Rice Biology, Zhejiang University, Hangzhou, 310029 People’s Republic of China; Institute of Plant Protection, State Key Laboratory for Biology of Plant Diseases and Insect Pests, Chinese Academy of Agricultural Sciences, Beijing, 100193 People’s Republic of China

**Keywords:** *Citrus limon*, Viroid, Small RNA, RNA interference

## Abstract

**Background:**

The advent of next generation sequencing technology has allowed for significant advances in plant virus discovery, particularly for identification of covert viruses and previously undescribed viruses. The *Citrus limon Burm. f.* (*C. limon*) is a small evergreen tree native to Asia, and . China is the world’s top lemon-producing nation.

**Findings:**

In this work, lemon samples were collected from southwestern of China, where an unknown disease outbreak had caused huge losses in the lemon production industry. Using high-throughput pyrosequencing and the assembly of small RNAs, we showed that the *Hop stunt viroid* (HSVd) was present in *C. limon* leaf sample. The majority of it is a main lemon producing agricultural cultivar*Hop stunt viroid* derived siRNAs (HSVd-siRNAs) in *C. limon* were 21 nucleotides in length, and nearly equal amount of HSVd-siRNAs originated from the plus-genomic RNA strand as from the complementary strand. A bias of HSVd-siRNAs toward sequences beginning with a 5′-Guanine was observed. Furthermore, hotspot analysis showed that a large amount of HSVd-siRNAs derived from the central and variant domains of the HSVd genome.

**Conclusions:**

Our results suggest that *C. limon* could set up a small RNA-mediated gene silencing response to *Hop stunt viroid*, Interestingly, based on bioinformatics analysis, our results also suggest that the large amounts of HSVd-siRNAs from central and variant domains might be involved in interference with host gene expression and affect symptom development*.*

## Findings

Viroids are autonomously replicating plant-specific pathogens that consist of a single-stranded, highly complementary, circular RNA without capsids or any detectable translational open reading frame (ORF). The *Hop stunt viroid* (HSVd), a member of the *Hostuviroid* genus within the *Pospiviroidae* family, is a typical viroid species that infects the common hop plants, citrus plants, and grapevines. The genome of HSVd contains five structural domains: two terminal regions, left (TL) and right (TR), pathogenic (P), variable (V), and central (C) domains, with a central conserved region (CCR) [1].

RNA silencing is a common antiviral mechanism in diverse eukaryotic hosts, and is also reported to be effective in defense against viroids in several plants [2–5]. Virus/viroid-derived small interfering RNAs (vsiRNAs) generated during this process were found to overlap with each other in sequence and can be assembled back into long contigs of the invading viral/viroid genome [5]. Based on this principle, our former work has proposed that the deep sequencing approach, combined with bioinformatics analysis, can be used to identify viruses through the assembly of virus derived small RNAs [6]. This approach provides a method to detect and identify covert viruses and previously undescribed virus groups during routine diagnosis, particularly for viruses with extremely low titer or without any prior pathogenic information. Here, we found that presence of HSVd-derived small RNAs (HSVd-siRNAs) in *C. limon*, suggesting that HSVd could infect *C. limon* and potentially be a pathogen leading to diseased *C. limon*, and we also supplied the high-resolution profiling of HSVd-siRNAs. The roles played by these small RNAs were discussed.

**Fig. 1:**
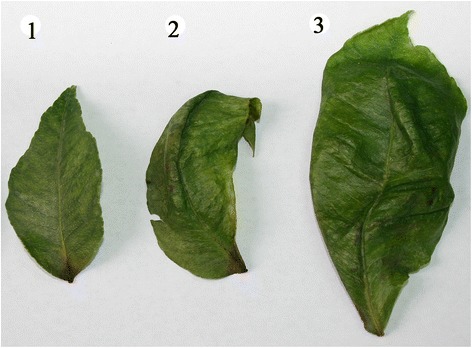
The symptom of *Citrus limon* leaves after infection with HSVd. Lanes 1–3 represent the symptom of different leaves from the same *Citrus limon* tree

**Fig. 2:**
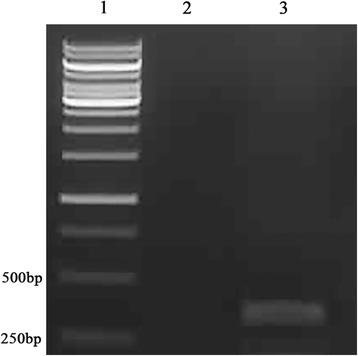
Reverse transcriptase PCR amplification of HSVd with a specific back-to-back primer. Lane 1, DNA ladder; Lane 2, healthy control; Lane 3, HSVd infected *Citrus limon* sample

*C. limon* leaf samples were collected in September 2012 from a lemon orchard in the city of DeHong, Yunnan province, China. The *C. limon* tree displayed stunting, leaf roll and mottle symptoms (Fig. 1), along with poor yield. Total RNAs were extracted using Trizol Reagent following the manufacturer’s instructions (Invitrogen, CA, USA). Total small RNAs ranging from 18 to 28 nucleotides (nt) were excised from 15 % polyacrylamide gel (PAGE) for ligation to 3′ and 5′ adaptors. After purification by electrophoresis, the final ligation products were reverse-transcribed and a cDNA library was constructed. After sequencing and trimming the adaptor sequences, 18–28 nt short reads were collected. The velvet program was chosen for genome assembly with 17 nucleotides as the minimal overlapping length (k-mer) required for joining two siRNAs into a contiguous sequence (contig) [5–7]. Assembly of 1.76 million vsiRNAs yielded 2613 contigs, including 535 contigs with lengths above 80 nt and 2078 with lengths below 80 nt. These assembled contigs were then aligned with the BLASTN program using the standard parameters in genome assembly (contigs with ≥90 % similarity). One long contig (294-nt), homologous to the nucleotide sequence of *Hop stunt viroid*-citrus strain with 100 % similarity (Accession No. FJ716188) was detected. This contig covered 97.35 % of the whole HSVd genome, lacking only the first 10 nucleotides. Subsequently, the existence of an intact HSVd genome in *C. limon* was verified by reverse transcriptase PCR using a pair of back-to-back primers (Fig. 2). (Forward primer: 5′-CCAACCTGCTTTTTGTCTATCTGAG-3′ and reverse primer: 5′-AAGACGAACCGAGAGGTGATGC-3′). The integrity of the amplified genome was verified by sequencing, and we found that the whole genome of our HSVd shared 100 % similarity with the HSVd CC-D isolate 1 (Accession No. FJ716188).

**Fig. 3:**
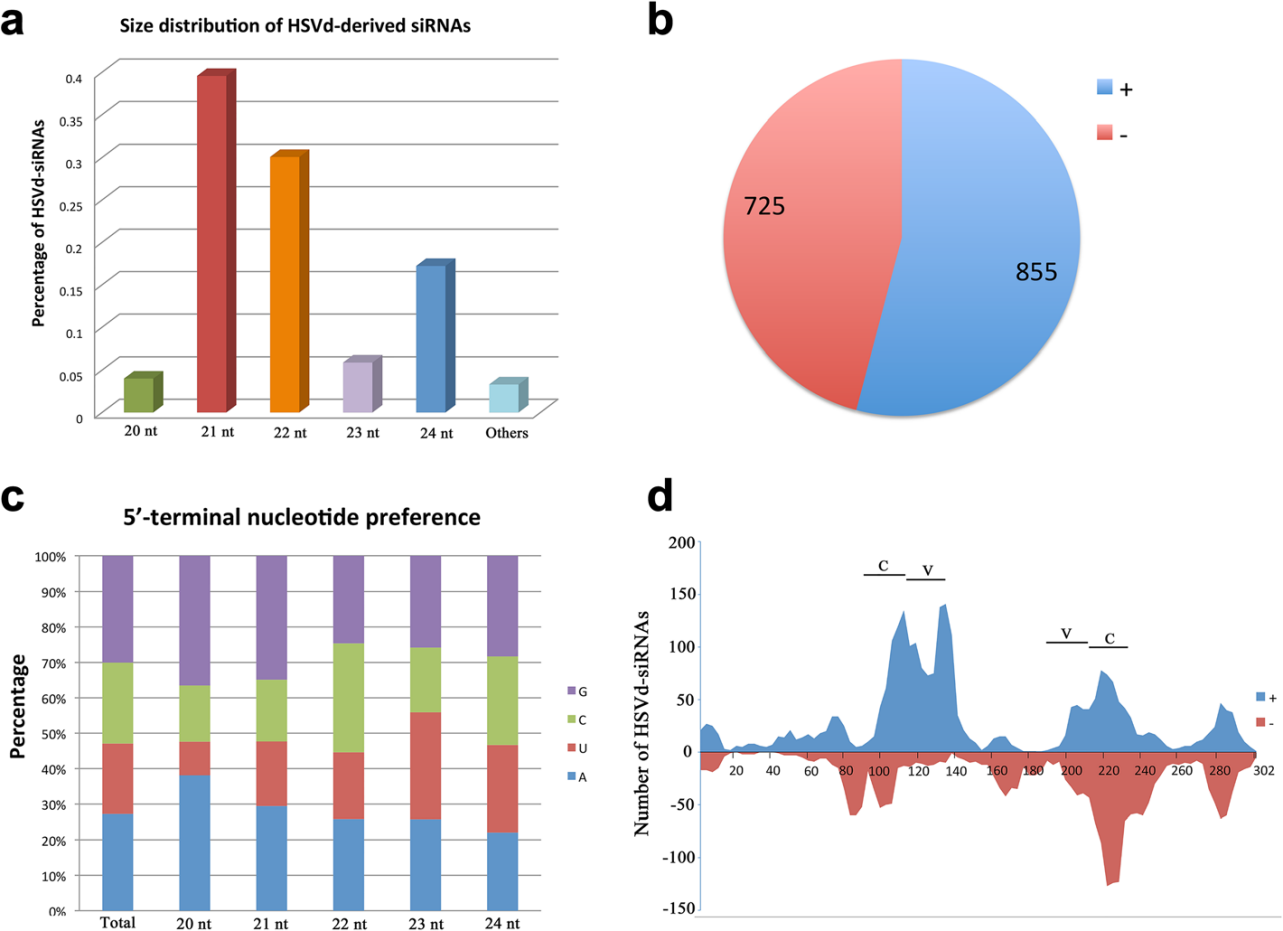
Profile of HSVd-siRNAs. 3**a**, Size distributions of vsiRNA sequences matching viroid genome. 3**b**, Statistical analysis of HSVd-siRNAs mapped to the genomic (+) or antigenomic (−) sequences. 3**c**, Relative frequency of 5′ terminal nucleotide, and the vsiRNAs were analyzed for both whole level (total) and different-sized species. 3**d**, Most HSVd-siRNAs derived from C (Central) and V (Variant) domains

To characterize and profile the HSVd-siRNAs along the viral genome, we aligned small RNA sequences with the HSVd genomic and antigenomic RNA sequences using bowtie tools and allowed zero mismatches [8]. The percentages of 20 to 24-nt HSVd-siRNAs identified in the viroid are shown in Fig. 3a; the 21-nt vsiRNAs predominate and account for almost 40 % of the total HSVd-siRNAs. 22-nt and 24-nt viroid-derived small RNAs account for 30.06 % and 17.22 % respectively. Our results are consistent with former reports that 21-nt vsiRNAs are predominant in viroid-infected plants [9, 10]. Furthermore, analysis of polarity distribution of the HSVd-siRNAs showed that HSVd-siRNAs were derived almost equally from the plus and minus strands of genome RNA (Fig. 3b), indicating that they might be produced from viroid replication intermediates during viroid replication by plant silencing machinery, and not by degradation of a plus-stranded viroid genomic RNA.

Previous studies have indicated that the 5′ terminal nucleotides of siRNAs have a pivotal role in directing the siRNAs to specific AGO complexes [11]. In contrast with the observations for diverse plant virus-specific small RNAs, which display a clear tendency to begin with Uracil (U) or Adenine (A) [12, 13], HSVd-siRNAs with a Guanine (G) at their 5′-end are the most abundant, and account for about 30.06 %, those with A, Cytosine (C) and U at their 5′-end are less recovered (27.22 %, 22.85 %, and 19.87 %, respectively) (Fig. 3c). To gain further insight into HSVd-siRNA biogenesis and sorting, the sequence complexity of these small RNAs was analyzed for different-sized species. For 21-nt HSVd-siRNAs, a clear preference for G and A as the 5′ terminal nucleotides is observed, however, a strong bias for sequences beginning with a 5′-C is observed in the 22-nt vd-siRNAs, indicative of sRNAs with high binding affinity for *Arabidopsis* AGO5-like Argonaute protein in *C. limon* (Fig. 3c). Our results are also consistent with former findings that 5′-C sRNAs in the AGO5 complex are mostly 22-nt in length in Arabidopsis [11]. Moreover, the role of AGO5 in RNA silencing has never been established and former works suggest that the loading of viroid derived sRNAs to AGOs with little or no intrinsic activity in the defense mechanism could contribute to the prevention of RISC-mediated degradation of viroid mature forms [1]. In addition, a bias for sequences beginning with a 5′-G was unexpected. 5′-G small RNAs were shown to be able to bind to *Arabidopsis* AGOs with less preference, and there have been no reports of 5′-G directing the siRNAs to specific AGO complexes [11]. The roles of this kind of small RNAs in the arm race between viroids and plants remain unclear, and we can’t rule out the possibility that *C. limon* could encode some other AGOs that interact with this class of small RNAs.

Additionally, single-base resolution maps of all redundant HSVd-siRNAs along with the genomes were created using bowtie tools and in-house Perl scripts [12]. Our results showed that HSVd-siRNAs could cover almost the entire viroid genome; however, there were two significant hotspots of small RNAs along the viroid genome, mainly localized in the regions between the end of central domain and variable domain (bases from 90 to 126, and from 185 to 220) (Fig. 3d). Approximately 82 % plus-and 76 % minus-strand HSVd-siRNAs mapped to only these two regions, indicating that certain regions of the viroid genome are preferentially targeted by dicers in *C. limon*. This result is consistent with a former work on HSVd infected grapevine, which found that most of the HSVd-siRNAs were also derived from variable (V) and central (C) domains [8]. Recent findings show that many viroid infections are associated with the appearance of viroid-specific small RNAs [9, 10, 14]. These RNAs have sizes similar to endogenous small interfering RNA and microRNA, and thus might alter normal gene expression in the host plants. Beatriz Navarro et al. found that two *Peach latent mosaic viroid*-derived small RNAs could target and cleave the mRNA encoding chloroplastic heat-shock protein 90 directly, causing albinism in peach leaves [15]. GomeZ et al. also found that HSVd-induced symptoms in plants are dependent on RDR6 activity, indicating that the involvement of the viroid-specific small RNAs may be associated with viroid pathogenesis [16]. Cachexia disease of citrus, with symptoms including discoloration, gumming and browning of phloem tissue, wood pitting, and bark cracking, is known to be determined by a “cachexia expression motif” of five to six nucleotides located in the variable domain [17]. Moreover, P. Serra et al.*,* found that a single nucleotide change in the “cachexia expression motif” could modulate citrus cachexia symptoms [18]. Our hypothesis is that the hotspot profiles of HSVd-siRNA from these domains may be related to pathogenic symptoms through regulation of *C. limon* gene expression. Therefore, based on the findings that sequence from No. 1 to 8 nt at the 5′-end of the miRNA has been observed to have a high degree of perfect complementariness to the target mRNA sequence [19], an algorithm was created with perl script and was used for selective extraction of the HSVd-siRNAs that allowed the first ten nucleotides at the 5′-end could cover the “cachexia expression motif” (loci 102–108, and loci 193–199). The 87 non-redundant HSVd-siRNAs were then used for target genes prediction by psRNATarget tools (cut-off threshold <2.0) [20], and the five target genes with highest value, as well as their related functions in symptom development, were listed in Table 1. The possibilities that these five genes might be targeted by its corresponding HSVd-siRNA were also validated by using other miRNA target prediction software (RNA22, data not shown) [21]. Therefore, testing whether HSVd-siRNAs may actually have any direct biological impact on expression of these genes could be an interesting aim for future studies.

**Table 1:** Predicted citrus mRNAs annotated as transcripts and targeted by HSVd-siRNAs

HSVd-siRNA No.	sRNA 5′-end^a^	Alignment^b^	Score^c^	Citrus transcript target^d^	Target predicted function
1	103	siRNA GAGAGAGGAUGCGGAGAGCGA	2.0	Orange1.1 g021690	E3 ubiquitin-protein ligase RING1-like
		**∶∶∶∶∶∶∶∶∶∶∶.∶∶∶∶∶.∶∶∶**			
		Target CUCUCUCCGACGUCUCUUGCU			
2	102	siRNA AGAGAGGAUGCGGAGAGCGAC	2.0	Cs5g11500.1	Cysteine--tRNA ligase
		**∶∶ ∶∶∶∶∶∶∶ ∶∶∶∶∶∶∶∶∶**			
		Target UCGCUCCUACACCUCUCGCUC			
3	198	siRNA UCUUCCAUUUUCUUCUUCCCU	2.0	TC24583	COP9 signalosome complex subunit 6a
		**∶∶∶∶∶∶ . . ∶∶∶∶∶∶∶∶∶∶∶∶**			
		Target AGAAGGAGGAAGAAGAAGGGA			
4	105	siRNA GGAGAGAGGAUGCGGAGAGC	2.0	Cs8g10955.1	S-adenosylmethionine synthase 3
		**∶∶∶∶∶∶∶∶∶ ∶∶∶∶∶∶∶∶∶**			
		Target CUCUCUCCGACGUCUCUUGCU			
5	199	siRNA CUUCCAUUUUCUUCUUCCCUG	2.0	EY709257	Pherophorin-C1 protein
		**∶∶∶ ∶∶∶∶∶∶∶∶∶∶∶∶∶∶∶∶**			
		Target GAACGUAAAAGAAGAAGGGAG			

^a^Location of HSVd-siRNA 5′ termini in the viroid genome

^b^Alignments between HSVd-siRNAs with predicted citrus mRNAs. HSVd-siRNAs were shaded in red

^c^The score of the complementarity between small RNA and their target transcript following the scoring schema of miRU by Zhang [22]

^d^Three *Citrus sinensis* database (transcript, cDNA, and Unigene) from the psRNATarget website were used for prediction

## Abbreviations

*C. limon*: *Citrus limon Burm. f.*
siRNA: small interfering RNA
HSVd: *Hop stunt viroid*
HSVd-siRNAs: *Hop stunt viroid* derived siRNAs
Contig: Contiguous sequence

## References

[1] Flores R, Gago-Zachert S, Serra P, Sanjuan R, Elena SF (2014). Viroids: survivors from the RNA world?. Annu Rev Microbiol.

[2] Ding SW, Li H, Lu R, Li F, Li WX (2004). RNA silencing: a conserved antiviral immunity of plants and animals. Virus Res.

[3] Sano T, Barba M, Li SF, Hadidi A (2010). Viroids and RNA silencing: mechanism, role in viroid pathogenicity and development of viroid-resistant plants. GM crops.

[4] Minoia S, Carbonell A, Di Serio F, Gisel A, Carrington JC, Navarro B (2014). Specific argonautes selectively bind small RNAs derived from potato spindle tuber viroid and attenuate viroid accumulation in vivo. J Virol.

[5] Wu Q, Luo Y, Lu R, Lau N, Lai EC, Li WX (2010). Virus discovery by deep sequencing and assembly of virus-derived small silencing RNAs. Proc Natl Acad Sci U S A.

[6] Xu Y, Huang L, Wang Z, Fu S, Che J, Qian Y (2014). Identification of Himetobi P virus in the small brown planthopper by deep sequencing and assembly of virus-derived small interfering RNAs. Virus Res.

[7] Zerbino D, Birney E (2008). Velvet: algorithms for de novo short read assembly using de Bruijn graphs. Genome Res.

[8] Langmead B, Trapnell C, Pop M, Salzberg SL (2009). Ultrafast and memory-efficient alignment of short DNA sequences to the human genome. Genome Biol.

[9] Navarro B, Pantaleo V, Gisel A, Moxon S, Dalmay T, Bisztray G (2009). Deep sequencing of viroid-derived small RNAs from grapevine provides new insights on the role of RNA silencing in plant-viroid interaction. Plos One.

[10] Giampetruzzi A, Roumi V, Roberto R, Malossini U, Yoshikawa N, La Notte P (2012). A new grapevine virus discovered by deep sequencing of virus- and viroid-derived small RNAs in Cv Pinot gris. Virus Res.

[11] Mi SJ, Cai T, Hu YG, Chen Y, Hodges E, Ni FR (2008). Sorting of small RNAs into Arabidopsis argonaute complexes is directed by the 5′ terminal nucleotide. Cell.

[12] Xu Y, Huang LZ, Fu S, Wu JX, Zhou XP. Population diversity of Rice stripe virus-derived siRNAs in three different hosts and RNAi-based antiviral immunity in *Laodelphgax striatellus*. Plos One. 2012;7. 10.1371/journal.pone.0046238 PMC3460854 23029445

[13] Donaire L, Wang Y, Gonzalez-Ibeas D, Mayer KF, Aranda MA, Llave C (2009). Deep-sequencing of plant viral small RNAs reveals effective and widespread targeting of viral genomes. Virology.

[14] Hammann C, Steger G (2012). Viroid-specific small RNA in plant disease. RNA Biol.

[15] Navarro B, Gisel A, Rodio ME, Delgado S, Flores R, Di Serio F (2012). Small RNAs containing the pathogenic determinant of a chloroplast-replicating viroid guide the degradation of a host mRNA as predicted by RNA silencing. Plant J.

[16] Gomez G, Martinez G, Pallas V (2008). Viroid-induced symptoms in Nicotiana benthamiana plants are dependent on RDR6 activity. Plant Physiol.

[17] Reanwarakorn K, Semancik JS (1998). Regulation of pathogenicity in hop stunt viroid-related group II citrus viroids. J Gen Virol.

[18] Serra P, Gago S, Duran-Vila N (2008). A single nucleotide change in Hop stunt viroid modulates citrus cachexia symptoms. Virus Res.

[19] Yue D, Liu H, Huang Y (2009). Survey of computational algorithms for microRNA target prediction. Curr Genomics.

[20] Dai X, Zhao PX (2011). psRNATarget: a plant small RNA target analysis server. Nucleic Acids Res.

[21] Miranda KC, Huynh T, Tay Y, Ang YS, Tam WL, Thomson AM (2006). A pattern-based method for the identification of microRNA binding sites and their corresponding heteroduplexes. Cell.

[22] Zhang Y (2005). miRU: an automated plant miRNA target prediction server. Nucleic Acids Res.

